# *Fusobacterium nucleatum* and its metabolite hydrogen sulfide alter gut microbiota composition and autophagy process and promote colorectal cancer progression

**DOI:** 10.1128/spectrum.02292-23

**Published:** 2023-10-27

**Authors:** Minyu Wang, Zheng Wang, Duncan James Lessing, Min Guo, Weihua Chu

**Affiliations:** 1 Department of Pharmaceutical Microbiology, School of Life Science and Technology, China Pharmaceutical University, Nanjing, China; 2 State Key Laboratory of Natural Medicines, China Pharmaceutical University, Nanjing, China; University of Nebraska-Lincoln, Lincoln, Nebraska, USA

**Keywords:** colorectal cancer, *Fusobacterium nucleatum*, hydrogen sulfide, autophagy, gut microbiota

## Abstract

*Fusobacterium nucleatum* is a harmful bacterium that produces hydrogen sulfide by metabolizing sulfur-containing amino acids, both of which have been shown to promote/initiate the development of various cancers, such as colorectal cancer, liver cancer, as well as lung cancer. However, few studies have reported how *F. nucleatum* acts on gut microbiota, influences the autophagy process, and promotes the development of colorectal cancer through the metabolite hydrogen sulfide. It was found that HCT116 cells secreted more inflammatory factors and showed higher survival as well as migration rate when given a pathological concentration of hydrogen sulfide. Using cellular transcriptome sequencing, it could be seen that after giving HCT116 cells *F. nucleatum* or *F. nucleatum* with L-cysteine, many inflammation-related signaling pathways, including autophagic pathways, were elevated compared to the control group. *In vivo*, the mice in the model groups displayed cancer progression, and the expression of some of the autophagy-related genes, *DRAM1*, *NBR1*, *ATG7*, *MAP1LC3A* were also increased in mice given *F. nucleatum* or *F. nucleatum* with L-cysteine when compared to the untreated control mice. The number of certain probiotic bacteria and fungi in the mice given *F. nucleatum* or *F. nucleatum* and L-cysteine decreased significantly; in contrast, the number of potentially pathogenic bacteria and fungi increased. These findings suggest that *F. nucleatum* and its metabolite hydrogen sulfide can modulate intestinal microbiota and autophagy as well as promote the development and progression of colorectal cancer.

**IMPORTANCE:**

Colorectal cancer (CRC) is the second most common cancer in the world; the main treatment for CRC is immunosuppressive therapy, but this therapy is only effective for a small percentage of CRC patients, so there is an urgent need for a treatment with fewer side effects and higher efficacy. This study demonstrated that *Fusobacterium nucleatum* with increased abundance in CRC can regulate the autophagy process and disrupt normal intestinal microbiota by producing hydrogen sulfide, factors that may be involved in the development and progression of CRC. This study may provide a reference for future CRC treatment options that are efficient and have fewer side effects.

## INTRODUCTION

Colorectal cancer (CRC) has become of increasing concern recently and is now noted as a high-risk cancer worldwide; it is the second most common cancer globally (1). The development of CRC is related to many factors, such as diet, genetic, gender, as well as ethnicity. It has been shown that chronic excessive alcohol consumption is closely associated with the development of many gastrointestinal cancers such as gastric cancer and CRC (2). In addition, the average diet found in developed countries, particularly those in the west, i.e., high intake of red meat, high oil, and fatty foods and insufficient intake of dietary fiber, has been shown as a risk factor in the development of these cancers particularly that of CRC (3). In addition, more than 80% of non-highly mutated CRC tumors carry mutations in the gene APC. This defect leads to a defect in the intestinal epithelial barrier, facilitating the invasion of inflammatory factors or pathogenic bacteria (1). The development of CRC is inextricably linked to the composition of the intestinal microbiota, There are 10^14^ bacteria in the human intestine, with species up to 10^13^, especially in the large intestine where bacteria are most abundant and dense. *Actinobacteria*, *Firmicutes*, *Bacteroides*, and *Proteobacteria* together constitute the main flora of the human intestine under normal conditions. Weir et al. (4) observed the enrichment of *Akkermansia muciniphila* in fecal samples from CRC patients. At the same time, another study reported higher levels of *Fusobacterium* and *Porphyromonas* and lower levels of *Ruminococcus* in fecal samples obtained from individuals suffering from CRC (5). A study of 285 patients with CRC showed that patients had the most significant decrease in fecal *Clostridiales* and a relative decrease in other butyrate-producing bacteria compared to healthy individuals (6); in the adenoma-carcinoma sequence, the levels of opportunistic pathogens such as *Fusobacterium nucleatum* (Fn), *Streptococcus*, and *Enterococcus* gradually increased, while the levels of butyrate-producing bacteria, such as *Bacteriodes* and *Clostridium*, gradually decreased. This loss of intestinal microbial diversity is known as dysbiosis. it is associated with chronic health conditions and cancer, as well as poor outcomes from some forms of cancer treatment. CRC can persist for many years without symptoms (7); thus, early detection and treatment are imperative for the patient’s survival.

Fn is a specialized anaerobic, gram-negative bacterium of the phylum *Fusobacteriota*, mainly colonizing the oral cavity, with five subtypes. Even if the bacterium is an oral resident, it may enter the intestinal tissues through the digestive tract or bloodstream and exert pro-inflammatory and cytotoxic effects through lipopolysaccharide (LPS), keratin-7 antisense (KRT7-AS), miRNA, the adhesins FadA, Fap2 (8), hydrogen sulfide (H_2_S), and alteration of intestinal microbiota to resulting in the development of diseases, such as CRC. Although Fn has been reported to enhance the effect of anti-programmed cell death protein (PD-L1) monoclonal antibody (mAb) in CRC, thus exerting an anti-tumor effect (9), most studies report the pro-cancer effect of Fn in CRC, namely Fn plays the role of “driver” in CRC (10), and several human samples currently investigating Fn include tissue, mucosa, stool, serum, and oral samples.

H_2_S in the intestine originates from the reduction reaction of sulfates in food and the metabolism of related substances, such as sulfur-containing amino acids and taurine. H_2_S prevents the oxidation of butyric acid and disrupts the colonic epithelial cell barrier, damaging non-transformed human cell lines and inducing free radical-related DNA damage. However, the physiological concentration of H_2_S has a protective and regulatory effect, which is called the H_2_S paradox, i.e., the role of H_2_S depends on its concentration. It has previously been shown that H_2_S is increased in fecal samples obtained from CRC patients.

Autophagy is a cellular response to external stimuli, which consists of four steps: formation of autophagic vesicles, extension and closure of autophagic membranes, a fusion of autophagic vesicles and lysosomes, and degradation of substances within autophagic lysosomes (11). It is inextricably linked to tumorigenesis and progression of tumor growth; in addition, it is associated with cellular senescence (12). Fn may promote tumor metastasis by activating the autophagic pathway; it accomplishes this through its metabolite H_2_S which has been reported to promote autophagy in hepatocellular carcinoma cells by inhibiting the phosphoinositide 3-kinase/serine/threonine kinase/mammalian target of rapamycin (PI3K/Akt/mTOR) signaling pathway (13). There are several reports reporting the promotion of CRC by Fn through its metabolite H_2_S and regulation of intestinal microbiota. For example, Fn can synthesize H_2_S from L-cysteine (Cys) and promote the proliferation of cancer cells (14). Reduced abundance of butyrate-producing bacteria and increased abundance of Fn were found in the intestinal lumen of rodents with CRC compared to healthy animals (15). In this study, it is demonstrated that Fn promotes autophagy in mice through its metabolite H_2_S. Simultaneously, it reduced the number of beneficial bacteria and increased the number of harmful bacteria, which may in turn promote CRC.

## RESULTS

### *F. nucleatum* could produce H_2_S by metabolizing L-cysteine

**Fig 1:**
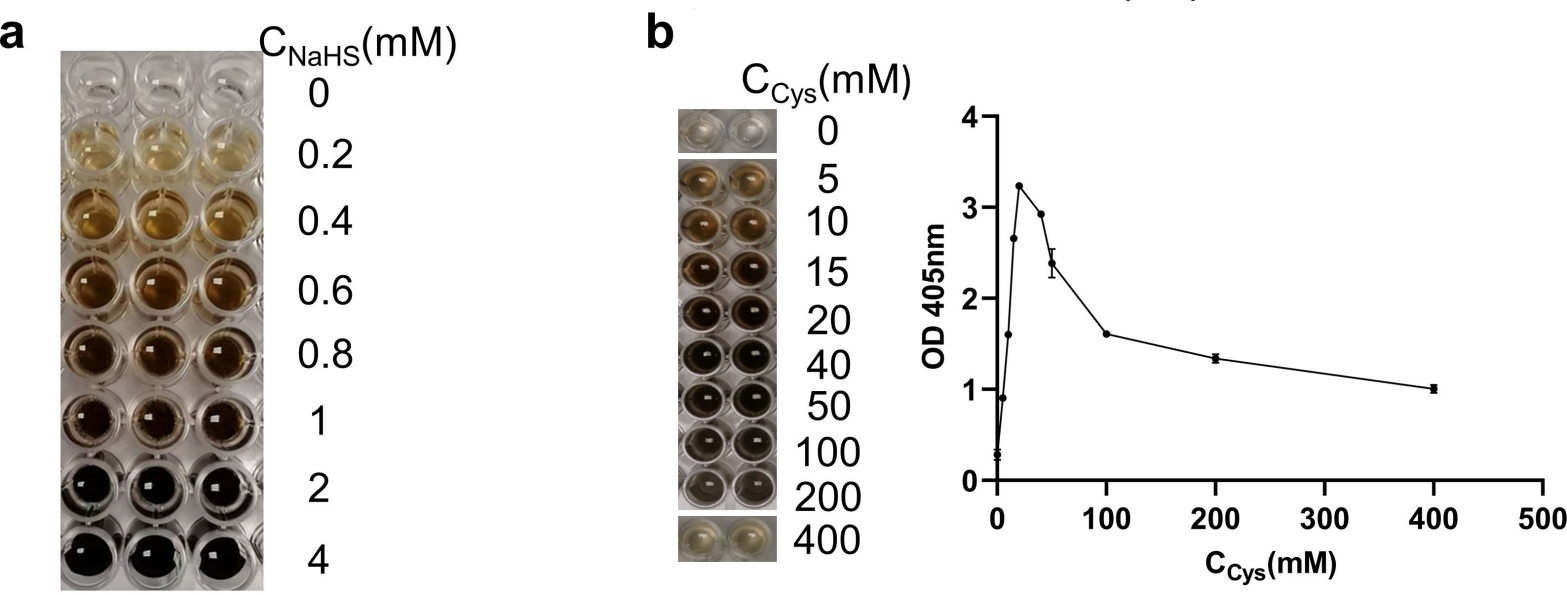
The H_2_S production capacity of Fn. (a) The detection of H_2_S corresponding to gradient NaHS concentrations (0, 0.2, 0.4, 0.6, 0.8, 1, 2, 4 mM from top to bottom) was measured by the bismuth chloride method. (b) The amount of H_2_S produced by bacteria was measured by the bismuth chloride method after administering Fn (10^9^ CFU) gradient concentrations (0, 5, 10, 15, 20, 40, 50, 100, 200, 400 mM) of L-cysteine.

From Fig. 1a, it can be seen that the color of the bismuth chloride precipitate deepened as the concentration of H_2_S increased. After culturing Fn with different concentrations of L-cysteine, the amount of H_2_S produced by Fn metabolism increased at first and thereafter decreased with an increasing concentration of Cys given, and when the concentration of Cys was 20 mM, the concentration of H_2_S produced by Fn changed from increase to decrease (Fig. 1b).

### Different concentrations of H_2_S had different effects on the proliferation and migration ability of different colon cancer cell lines

**Fig 2:**
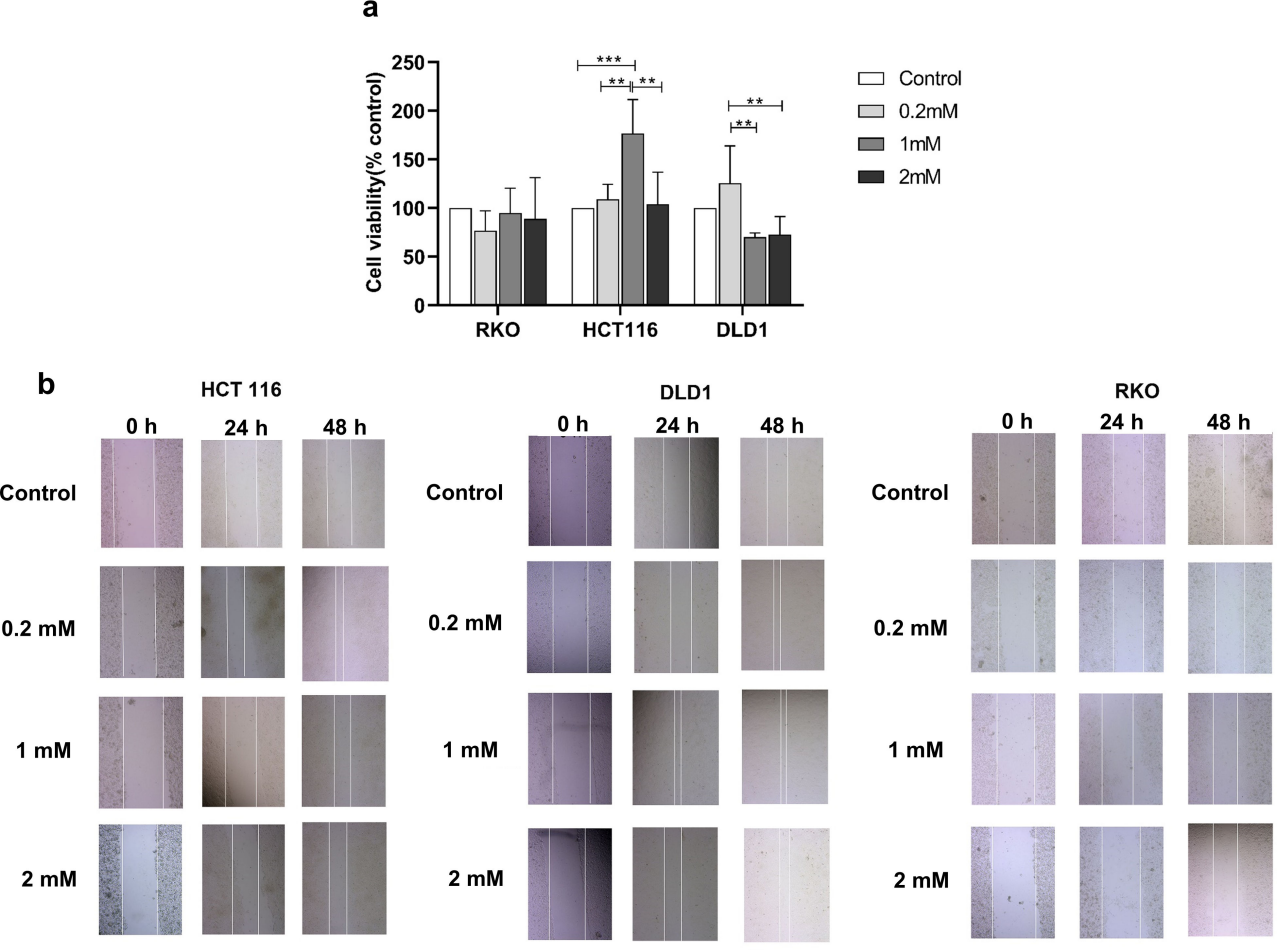
Effects of different concentrations of H_2_S on the survival and migration rate of three human-derived colon cancer cells. (a) Three colon cancer cell lines, RKO, HCT116, and DLD1, were given different concentrations of sodium hydrosulfide, and the survival rate of cells was determined by the CCK-8 method after 24 h. Data are presented as the mean ± SD, **P < 0.01, ***P < 0.001 (one-way analysis of variance). (b) Three colon cancer cell lines, RKO, HCT116, and DLD1, were given different concentrations of sodium hydrosulfide and scratched with a sterile tip, and the changes in the width of the scratches were observed at 0, 24, and 48 h, respectively.

The effect of H_2_S at different concentrations on the survival rate and the migration rate of three colon cancer cell lines was investigated. As shown in Fig. 2a, the relative survival rate of cells increased significantly when compared with the blank group only when HCT116 cells were given 1 mM of sodium hydrosulfide. In contrast, the survival rates of RKO and DLD1 did not change significantly when compared with the control group. The cell scratch assay (Fig. 2b) also demonstrated a significant narrowing of the width of the scratch at 48 h after administration of 1 mM sodium hydrosulfide to HCT116 cells. In addition, the width of the scratch also significantly narrowed at 48 h after administration of 0.2 mM sodium hydrosulfide to HCT116. For DLD1 cells, 0.2 mM and 1 mM sodium hydrosulfide significantly promoted the migration rate of the cells; however, there was no significant increase in the migration rate of RKO cells regardless of which concentration of sodium hydrosulfide was given. Because in the Cell Counting Kit-8 (CCK8) assay, when colon cancer cells were given different concentrations of sodium hydrosulfide, only HCT116 showed a significant increase in survival rate when the concentration of sodium hydrosulfide was 1 mM compared with the blank control group, while the survival rate of the other two types of cells did not change significantly compared with the blank control group; it was decided to use HCT116 as the subject of the follow-up experiment.

### Pathological concentrations of H_2_S promoted the secretion of inflammatory factors of HCT116 in colon cancer cells

**Fig 3:**
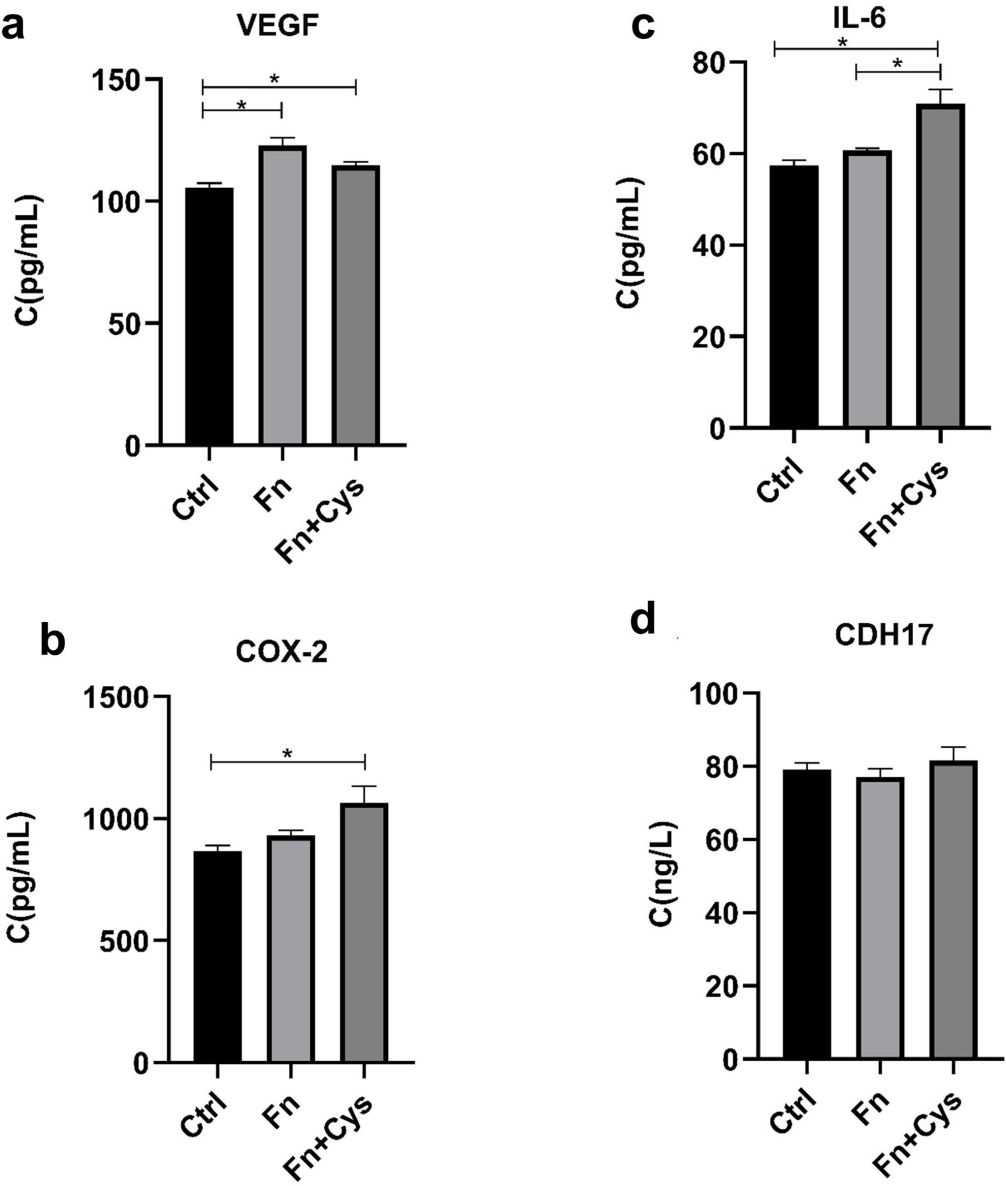
H_2_S at the same concentration as H_2_S in the intestines of CRC patients promoted the pro-inflammatory effect of colon cancer cells HCT116. The expression levels of inflammatory factors (a) VEGF, (b) COX-2, (c) IL-6, and (d) CDH17 in the cell supernatants were measured after co-culturing HCT116 with Fn or with Fn and Cys. Data are presented as the mean ± SD, *P < 0.05 (one-way analysis of variance).

Fn (10^9^ CFU) was able to metabolize 20 mM Cys to produce pathological changes in HCT116 cells. The supernatant of the cell culture was taken after 4 h co-culture with Fn, and the expression of inflammatory factors were detected by enzyme linked immunosorbent assay (ELISA). The results showed that the expression levels of inflammatory factors vascular endothelial growth factor (VEGF), interleukin-6 (IL-6), and cyclooxygenase 2 (COX-2) were significantly increased after the addition of Cys when compared with the control group (Fig. 3a through c), but the expression of CDH17 was not significantly altered (Fig. 3d). This suggests that colonic concentration of H_2_S in CRC patients can promote the secretion of inflammatory factors of HCT116.

### *F. nucleatum* and H_2_S alter the transcriptome profile of HCT116

**Fig 4:**
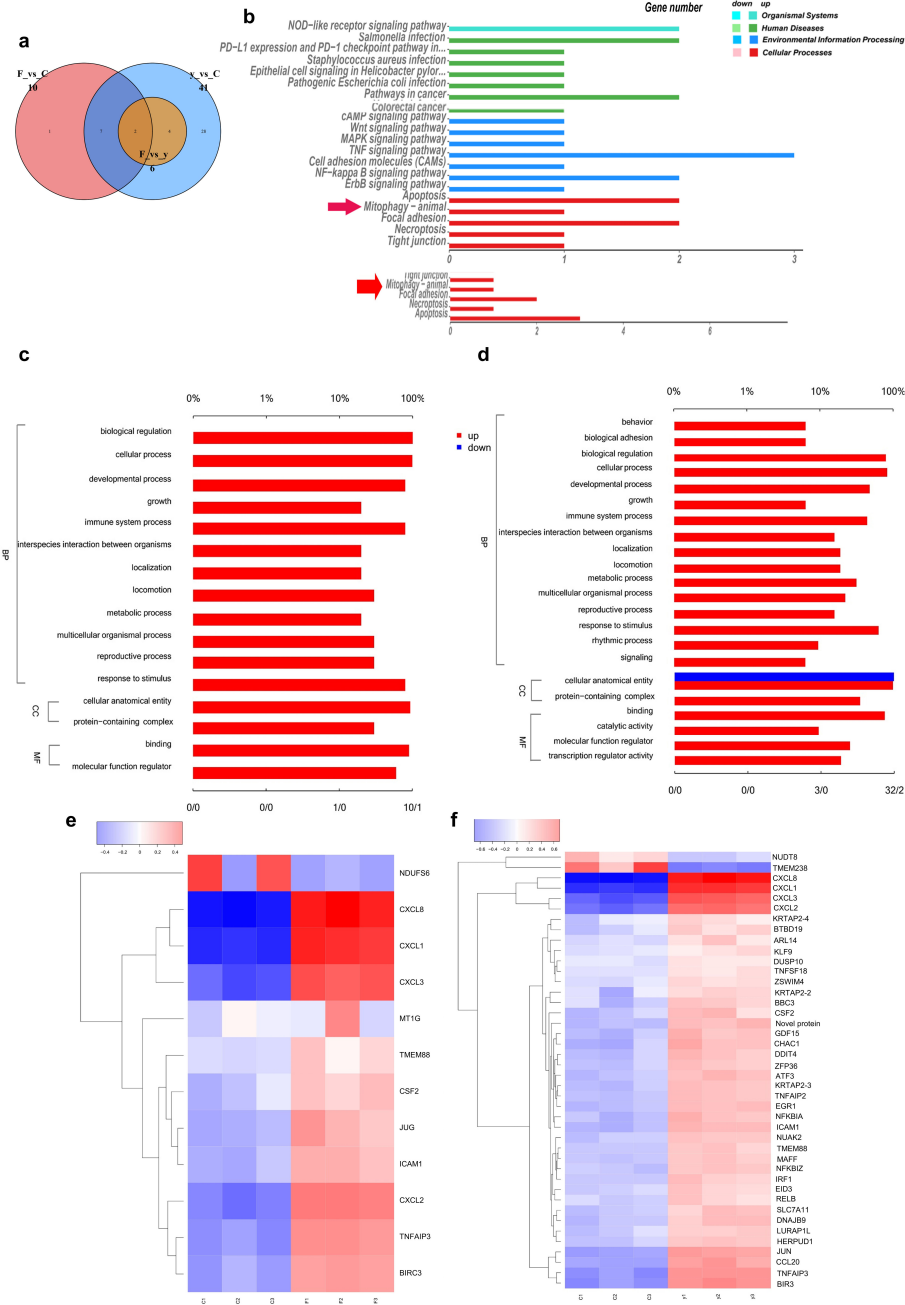
Changes in cell transcriptome profile after administration of Fn or Fn with Cys to HCT116 cells. (a) Venn diagram of genes differentially expressed between two groups, C means HCT116 control group, F means CRC + Fn group, and y means CRC + Fn + Cys group. Part of the enriched Kyoto Encyclopedia of Gene and Genomes pathways and Gene Ontology terms between the HCT116 + Fn group and HCT116 group (b top and c), HCT116 + Fn + Cys group and HCT116 group (b bottom and d). Heat map of differences in gene expression between HCT116 + Fn group and HCT116 group (e), HCT116 + Fn + Cys group and HCT116 group (f).

To investigate the response of Fn and H_2_S in cancerous human cells and the effect they have on these cells, the alteration of the transcriptome profile in human colon cancer cells was analyzed. From Fig. 4a, it can be seen that nine genes differentially expressed between the HCT116 + Fn group and the HCT116 group overlapped with the genes differentially expressed between the HCT116 group and the HCT116 + Fn + Cys group. These nine genes included *JUN*, *BIRC3*, *ICAM1*, *CXCL8*, *CXCL1*, *CXCL3*, *CXCL2*, *CSF2*, and *TNFAIP3*, which are all pro-oncogenes and pro-inflammatory genes. The Venn diagram shows that the genes with different expression between the HCT116 + Fn group and the HCT116 + Fn + Cys group are *TMEM238*, *CCL20, DNAJB9*, etc. Moreover, the HCT116 + Fn + Cys group had far more differentially expressed genes when compared with the HCT116 group than the HCT116 + Fn group when compared with the HCT116 group, further suggesting that Fn plays a role in promoting cancer development, and H_2_S further enhanced the expression of pro-oncogenes.

Next, in order to investigate the effect of Fn and H_2_S on the gene pathway of cancer cells, a Kyoto Encyclopedia of Genes and Genomes (KEGG) pathway analysis was performed for the HCT116 + Fn group and HCT116 group, then subsequently the HCT116 + Fn + Cys group and HCT116 group.a When HCT116 + Fn was compared with the HCT116 group (Fig. 4b top), in addition to the upregulation of mitophagy, a number of other abnormalities were noted. These include the pathways as pathway related to autophagy, nucleotide oligomerization domain (NOD)-like receptor signaling pathway in the organismal system; human T-cell leukemia virus 1 infection, advanced glycation end-products-receptor for advanced glycation end products (AGE-RAGE) signaling pathway in diabetic complication; salmonella infection, pathways in cancer in the human disease; ubiquitin-mediated proteolysis in the genetic information processing; tumor necrosis factor (TNF) signaling pathway, nuclear factor kappa-B (NF-κB) signaling pathway in the environmental information processing; apoptosis, focal adhesion in the cellular processes were all up-regulated; oxidative phosphorylation, metabolic pathway were down-regulated in the HCT116 + Fn group. When HCT116 + Fn + Cys was compared with HCT116 group (Fig. 4b bottom), NOD-like receptor signaling pathway, AGE-RAGE signaling pathway in diabetic complication, human T-cell leukemia virus 1 infection, salmonella infection, TNF signaling pathway and NF-κB signaling pathway, apoptosis and focal adhesion and mitophagy were all up-regulated in HCT116 + Fn + Cys group. In addition, H_2_S also up-regulated mitogen-activated protein kinase (MAPK) and cyclic AMP (cAMP) signaling pathways and enhanced iron death of cells. These results suggested that H_2_S enhanced the induction of Fn on relevant cancer signaling pathways, altered the body’s immune effects against viruses and bacteria, and promoted autophagy and apoptosis cellular processes.

There are three branches of Gene Ontology (GO), the GO terms enriched between the HCT116 + Fn group and the HCT116 group (Fig. 4c) are 12 belonging to BP (biological process), mainly focusing on cellular process, immune system process, biological regulation, and response to a stimulus; two terms belonged to CC (cellular component), and the variation focused on cellular, anatomical entity; two terms belonged to MF (molecular function), and the main focus was on binding. The enriched GO terms (Fig. 4d) between the HCT116 + Fn + Cys group and the HCT116 group were 16 belonging to BP, again mainly focused on the above processes, and four belonging to MF, mainly focused on binding, molecular function regulator, and transcription regulator activity.

To further investigate the significance of up- or down-regulated expression of genes between the different groups, the heat map of gene expression was plotted. Ten genes were significantly up-regulated in the HCT116 + Fn group when compared with the HCT116 group (Fig. 4e), including *CXCL8*, *CXCL1*, *CXCL3*, *CXCL2*, *TNFAIP3*, *ICAM1*, etc. The most significantly down-regulated gene was *NDUFS6*. In the HCT116 + Fn + Cys group, when compared with the HCT116 group (Fig. 4f), 40 individual genes were among some of the most significantly up-regulated genes. These included but are not limited to *CXCL8*, *CXCL1*, *CXCL3*, *CXCL2*, *TNFAIP3*, and *ICAM1*, in addition to *ATF3*, *TNFSF18*, *TNFAIP2*, *GDF15*, *DDIT4*, which are all related to inflammation, apoptosis, and DNA damage. In addition, *EGR1* can regulate macrophages, *NFKBIA* is related to apoptosis, and *TMEM88* is a membrane transporter protein.

Similarly, the top 20 genes with the most significant expression differences (up- or down-regulated) between the different groups were tabulated (Tables 1 and 2). Among the top 10 genes with up-regulated expression between the two groups (HCT116 + Fn group and HCT116 + Fn + Cys group), four genes were up-regulated in both groups, and among the top 10 genes with down-regulated expression in the two groups, there were no genes with down-regulated expression in both groups.

**TABLE 1:** Top 20 significantly differentially expressed genes of HCT116 cultured with Fn for 4 h

	Gene name	Description
Up-regulated	TNF	Tumor necrosis factor
	ARMH2	Armadillo-like helical domain containing 2
	C8orf89	Chromosome 8 open reading frame 89
	ACTC1	Actin alpha cardiac muscle 1
	MAP1LC3A	Microtubule-associated protein 1 light chain 3 alpha
	PCP4	Purkinje cell protein 4
	OR2A2	Olfactory receptor family 2 subfamily A member 2
	NACA2	Nascent polypeptide-associated complex subunit alpha 2
	SLAMF9	SLAM family member 9
	IL13RA2	Interleukin-13 receptor subunit alpha 2
Down-regulated	CLDN24	Claudin 24
	MT1B	Metallothionein-1B
	CELA3A	Chymotrypsin-like elastase 3A
	PPP1R14A	Protein phosphatase 1 regulatory inhibitor subunit 14A
	CRIP3	Cysteine-rich protein 3
	TAS2R30	Taste 2 receptor member 30
	CD1D	CD1d molecule
	SMIM11B	Small integral membrane protein 11B
	GRIA3	Glutamate ionotropic receptor AMPA type subunit 3
	CD8B	CD8b molecule

**TABLE 2:** Top 20 significantly differentially expressed genes of HCT116 cultured with Fn and L-cysteine for 4 h

	Gene name	Description
Up-regulated	TNF	Tumor necrosis factor
	SELE	Selectin E
	NACA2	Nascent polypeptide-associated complex subunit alpha 2
	CCL20	C-C motif chemokine ligand 20
	WFDC10A	WAP four-disulfide core domain 10A
	MAP1LC3A	Microtubule-associated protein 1 light chain 3 alpha
	ARMH2	An armadillo-like helical domain containing 2
	RFPL1	Ret finger protein-like 1
	CXCL11	C-X-C motif chemokine ligand 11
	FAM170A	Family with sequence similarity 170 members A
Down-regulated	SPRR2E	Small proline-rich protein 2E
	SFTPD	Surfactant protein D
	FAM24A	Family with sequence similarity of 24 members A
	LCE1F	Late cornified envelope 1F
	C19orf84	Chromosome 19 open reading frame 84
	MS4A12	Membrane-spanning 4-domains A12
	GJD4	Gap junction protein delta 4
	AWAT2	Acyl-CoA wax alcohol acyltransferase 2
	PLEK	Pleckstrin
	SMIM11B	Small integral membrane protein 11B

It has been shown that Fn may promote CRC metastasis by activating the autophagic pathway, and H_2_S may promote autophagy in hepatocellular carcinoma cells by inhibiting the PI3K/Akt/mTOR signaling pathway (13). Besides, there is little literature on whether Fn promotes autophagy through the production of hydrogen sulfide and the association between autophagy and CRC, so we chose the mitophagy pathway rather than the other pathways in KEGG pathway analysis. However, mitophagy only represents the autophagy of mitochondria, whereas autophagy would have a broader scope, including but not limited to mitophagy, and we wanted to explore a more generalized level of autophagy. As such, this study attempted to search for autophagy-related genes. In the HCT116 + Fn group, the autophagy-related genes that increased or decreased when compared with the HCT116 group were tabulated in Table 3. In the HCT116 + Fn + Cys group, the autophagy-related genes that increased or decreased when compared with the HCT116 group became more numerous, they were also tabulated in Table 4. Genes were selected based on expression that was either co-up- or down-regulated in two groups, namely *MAP1LC3A*, *DRAM1*, *NBR1*, *ATG7,* and *ATG16L1* for quantitative real-time PCR (RT-qPCR) in order to observe the role played by these autophagy-related genes in CRC.

**TABLE 3:** Autophagy-related genes that increased or decreased in the HCT116 + Fn group compared with the HCT116 group

	Gene name	Description
Up-regulated	DEPP1	DEPP1 autophagy regulator
	ATG16L2	Autophagy-related 16 like 2
	DRAM1	DNA damage-regulated autophagy modulator 1
	ULK1	Unc-51-like autophagy-activating kinase 1
	NBR1	NBR1 autophagy cargo receptor
	SOGA1	Suppressor of glucose, autophagy-associated 1
	ATG7	Autophagy-related 7
	ATG10	Autophagy-related 10
Down-regulated	EI24	EI24 autophagy-associated transmembrane protein
	ATG101	Autophagy-related 101
	EPG5	Ectopic P-granules autophagy protein 5 homolog
	ATG16L1	Autophagy-related 16 like 1
	ATG3	Autophagy-related 3

**TABLE 4:** Autophagy-related genes that increased or decreased in the HCT116 + Fn + Cys group compared with the HCT116 group

	Gene name	Description
Up-regulated	ELAPOR1	Endosome-lysosome-associated apoptosis and autophagy regulator 1
	DRAM2	DNA damage-regulated autophagy modulator 2
	DEPP1	DEPP1 autophagy regulator
	AMBRA1	Autophagy and beclin 1 regulator 1
	ATG13	Autophagy-related 13
	ATG2A	Autophagy-related 2A
	ATG16L2	Autophagy-related 16-like 2
	DRAM1	DNA damage-regulated autophagy modulator 1
	ULK1	Unc-51-like autophagy-activating kinase 1
	ATG14	Autophagy-related 14
	ULK2	Unc-51-like autophagy-activating kinase 2
	NBR1	NBR1 autophagy cargo receptor
	EPG5	Ectopic P-granules autophagy protein 5 homolog
	ATG9A	Autophagy-related 9A
	ATG7	Autophagy-related 7
	ATG3	Autophagy-related 3
	RUBCN	Rubicon autophagy regulator
	ATG12	Autophagy-related 12
	ATG5	Autophagy-related 5
	ELAPOR2	Endosome-lysosome-associated apoptosis and autophagy regulator family member 2
Down-regulated	ATG4C	Autophagy-related 4C cysteine peptidase
	EI24	EI24 autophagy-associated transmembrane protein
	ATG16L1	Autophagy-related 16-like 1
	ATG4B	Autophagy-related 4B cysteine peptidase
	SOGA1	Suppressor of glucose, autophagy-associated 1
	FYCO1	FYVE and coiled-coil domain autophagy adaptor 1
	ATG9B	Autophagy-related 9B

Taken together, the results suggest that Fn can alter the transcriptome of cancer cells, including autophagy-related genes, and further induced oncogenic factors and pathways through its metabolite H_2_S.

### *F. nucleatum* and its metabolite H_2_S promoted intestinal cancer in azoxymethane (AOM)/dextran sulfate sodium (DSS) mice

**Fig 5:**
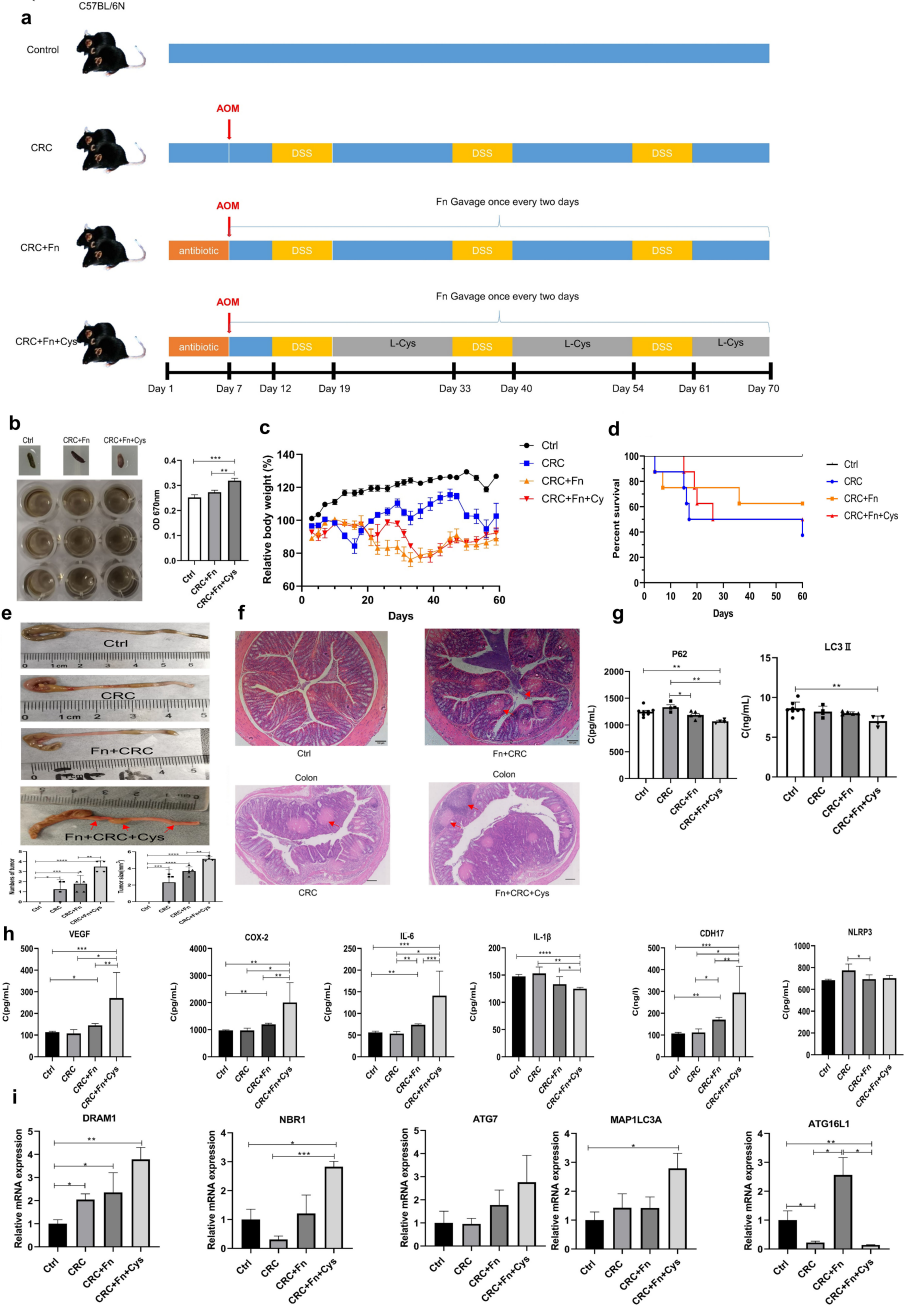
The mouse colorectal cancer model was created with AOM/DSS to observe the survival rate and the lesions of tissues of mice and to observe the expression of inflammatory factors and autophagy-related genes in mouse serum or colons by ELISA and RT-qPCR. The number of mice in each group was at least 3. (a) Schematic diagram of a mouse CRC model with AOM/DSS and the administration of bacteria and Cys. (b) The appearance of feces of mice and determination of the amount of H_2_S in feces. (c) Graphs of body weight changes in mice. (d) Survival curves of mice. (e) Colons of mice, the number and volume of the tumors in colons in each group. (f) H&E staining of the colons mice. Scale bar = 100 µm. (g) Determination of the expression levels of p62 and LC3II in the serum of mice by ELISA. Data are presented as the mean ± SEM, *P < 0.05, **P < 0.01 [one-way analysis of variance (ANOVA)]. (h) Expression levels of NLRP3, IL-1β in serum and VEGF, COX-2, IL-6, and CDH17 in colons of mice were determined by ELISA. Data are presented as the mean ± SEM, *P < 0.05, **P < 0.01, ***P < 0.001, ****P < 0.0001 (unpaired t-test). (i) Expression levels of autophagy-related genes in colons of mice were determined by RT-qPCR. Data are presented as the mean ± SEM, *P < 0.05, **P < 0.01, ***P < 0.001, ****P < 0.0001 (one-way ANOVA).

For further *in vivo* experiments, mice were divided into four groups: control group, CRC model group, CRC + Fn group, and CRC + Fn + Cys group (Fig. 5a), as detailed in the Materials and Methods section. In order to verify that the H_2_S in the mice did indeed increase after the administration of Fn and Cys to CRC mice, H_2_S in the feces of mice was measured. It was seen that H_2_S in the feces of mice given Fn and Cys increased when compared with that of the control and CRC + Fn groups (Fig. 5b). In addition, CRC + Fn groups and CRC + Fn + Cys group mice had traces of blood within their fecal matter. There was no significant difference of body weight between the mice in the CRC + Fn and CRC + Fn + Cys groups (Fig. 5c). The body weight of the mice in both groups, especially at the late modeling stage, was significantly lower than that of the control and CRC model groups. Combining the survival curves of the four groups of mice (Fig. 5d), the CRC + Fn and CRC + Fn + Cys groups, especially the latter group, had significantly lowered survival status when compared with the CRC + Fn groups. Then, the morphology of the colonic regions was observed (Fig. 5e) and it was observed that the CRC + Fn + Cys group had a significantly shorter colon length with cysts (marked by arrows) and more severe inflammation than that of the other groups. Besides, the number and size of tumors in the CRC + Fn + Cys group were larger than that of the CRC and CRC + Fn group. To further understand the lesions within the tissues, hematoxylin and eosin (H&E) staining was performed (Fig. 5f) on the colon tissues of each group. The results of which showed more cysts (pink circular plaques, marked by arrows) and shorter intestinal villi in the CRC + Fn group when compared to the control and CRC groups, while in the CRC + Fn + Cys group, there were noticeable structural changes of intestinal cavities, destruction of cupulae, and tumor invasion (marked by arrows) in the colons of the mice. Taken together, these results suggest that Fn and its metabolite H_2_S promoted intestinal cancer in AOM/DSS mice.

Combined with the results of cellular transcriptome sequencing, it was speculated that Fn and its metabolite H_2_S stimulated autophagy during the process of CRC. Autophagy markers P62 and LC3II in mouse serum were analyzed (Fig. 5g). It was found that there was a significant decrease in P62 in serum of mice in the CRC + Fn + Cys group when compared with the control group. Simultaneously, there was a significant decrease in LC3II in serum of mice in the CRC + Fn + Cys group when compared with the control group, which was contradictory to the decrease in P62 level, because it has been shown that during autophagy, the expression of P62 decreased while the expression of LC3II increased (16, 17). It is hypothesized that LC3II is degraded in the lumen of autophagic lysosomes at the end of autophagy, and excessive autophagy leads to a decrease in LC3II levels. These data are consistent with previous findings showing that Fn and H_2_S promoted the level of autophagy in mice or tumor cells (13, 14). In addition to the above, the expression levels of inflammatory factors VEGF, COX-2, IL-6, and CDH17 in the colonic tissues of mice in the CRC + Fn and CRC + Fn + Cys groups (Fig. 5h) were significantly higher than those of the control group, and the levels of inflammatory factors in the colonic tissues of mice in the CRC + Fn + Cys group were significantly higher than those in the CRC + Fn group, which again verified that Fn and/or its product H_2_S can promote the development of CRC. NLRP3, an inflammatory vesicle in mouse serum, did not differ significantly between the four groups. The expression of IL-1β in the CRC + Fn + Cys group was significantly lower than that of the other three groups in our experiments. This may be due to the fact that the secretion of IL-1β is dependent on NLRP3 activation.

Finally, RT-qPCR was used to verify the autophagy-related genes obtained by cell transcriptome sequencing. These genes included *DRAM1*, *NBR1*, *ATG7*, *MAP1LC3A*, and *ATG16L1*. As seen from the Fig. 5i, the expression of *DRAM1*, *NBR1*, *ATG7*, and *MAP1LC3A* genes increased gradually in both the CRC + Fn group and CRC + Fn + Cys group, especially *DRAM1*, *NBR1*, and *MAP1LC3A*, which showed a significant increase in expression in CRC + Fn + Cys group when compared with the control group. However, the expression level of *ATG16L1* in the CRC + Fn + Cys group was significantly lower than that of the control group. These results are consistent with the results obtained from cellular transcriptome sequencing in this study. These data suggest that Fn promotes autophagy through its metabolite H_2_S, which may be closely related to the inflammation and development of CRC.

### *F. nucleatum* and its metabolite H_2_S dysregulated intestinal microecology in mice

**Fig 6:**
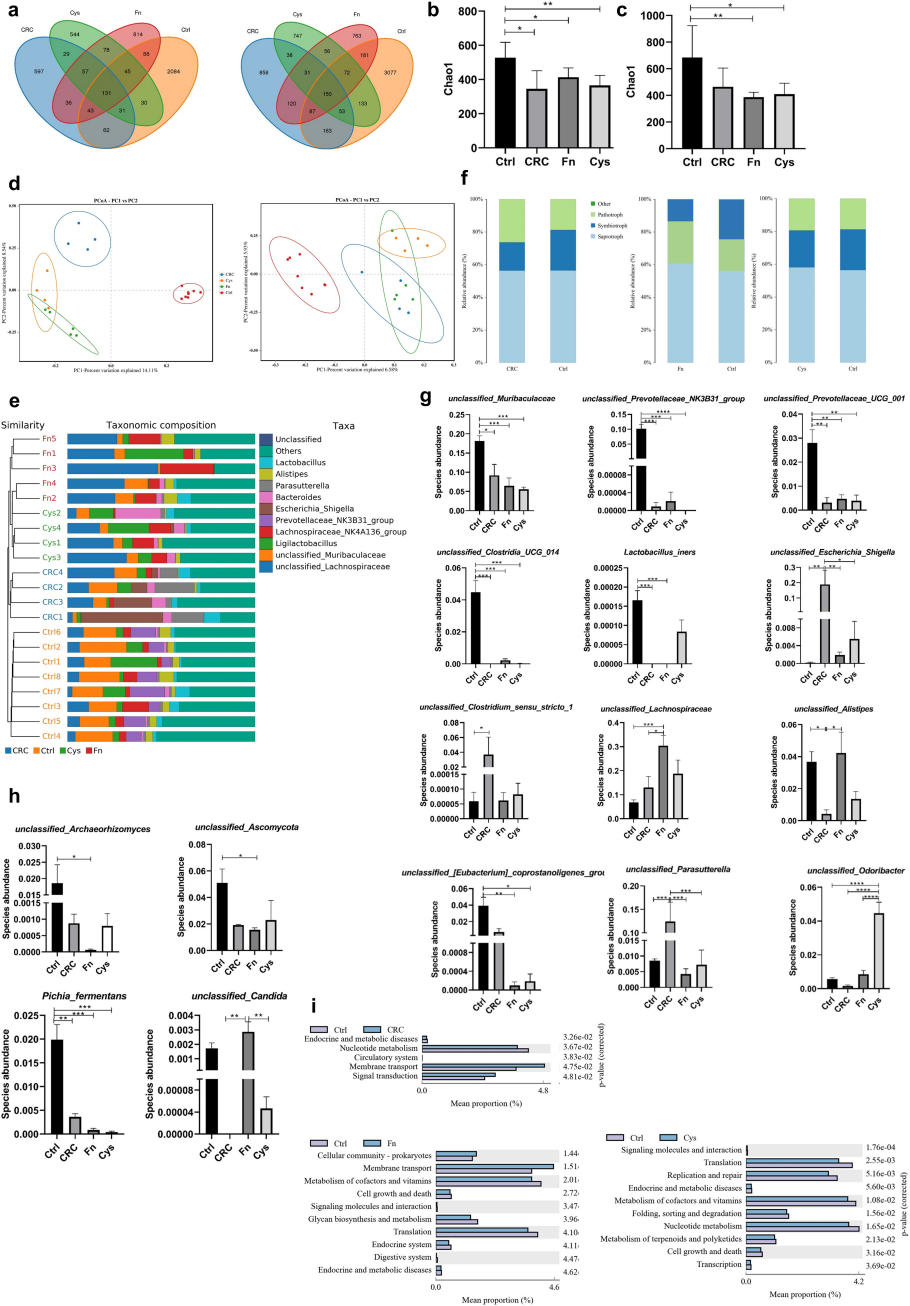
The alpha and beta diversity of bacteria and fungi were first compared between groups, respectively, followed by relative abundance of each bacterium or fungus between groups to compare the differences in composition and abundance of bacteria or fungi, respectively, and functional analysis of bacteria were used to compare the changes in signaling pathways between groups. The number of mice in the control group was 8, and the number of mice in the CRC and CRC + Fn + Cys group was 4, respectively. The number of mice in the Fn group was 5. (a) Venn diagram of OTU distribution of bacteria (left) and fungi (right). Measures of bacterial (b) and fungal (c) alpha diversity, i.e., Chao1 index. (d) PCoA analysis of beta diversity of bacteria (left) and fungi (right). (e) Combined cluster number and histogram analysis of bacteria. (f) Distribution of different types of fungi (saprotroph, symbiotroph, and pathotroph) among different groups. Differences in abundance of bacteria between groups. (g) Differences in abundance of fungi between groups. (h) Plot of predicted PICRUSt2 COG function results for bacteria. Ctrl, CRC, Fn, Cys groups represent the control, CRC, CRC + Fn, CRC + Fn + Cys groups, respectively. Data are presented as the mean ± SEM, *P < 0.05, **P < 0.01, ***P < 0.001, ****P < 0.0001 (one-way analysis of variance).

**Fig 7:**
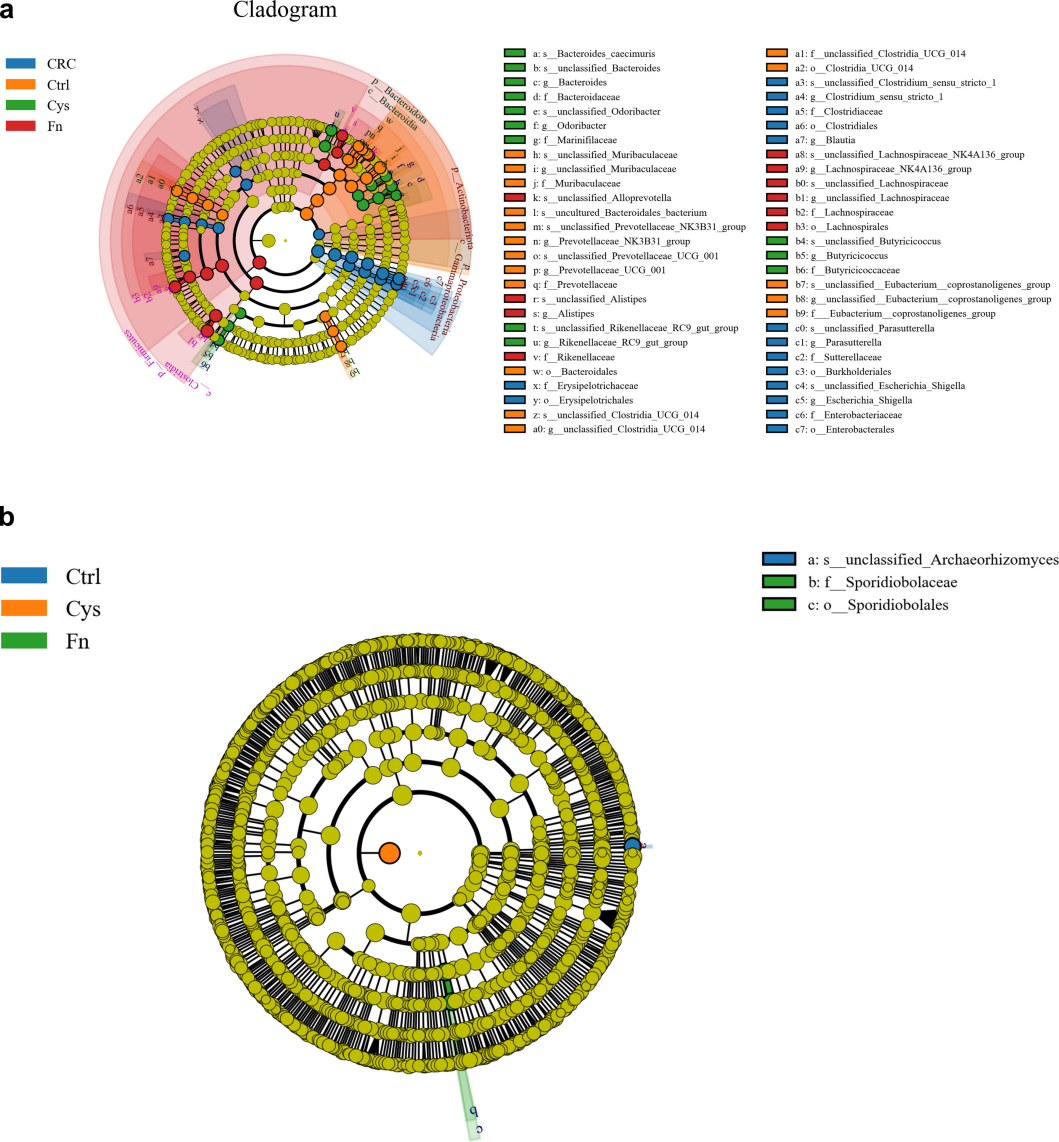
Evolutionary branching plots of LEfSe analysis for bacteria (a) and fungi (b).

Finally, the modulating effects of Fn and its metabolite, H_2_S on intestinal microbiota (bacteria and fungi) of mice, were investigated. By 16S rRNA and ITS sequencing of mouse feces, various indicators of alpha diversity and beta diversity of microbiota in the intestine of mice were obtained. Firstly, the Venn diagram showed that the number of operational taxonomic units (OTUs) unique to each group of bacteria (Fig. 6a, left) was much larger than the number of OTUs shared between groups, indicating that the composition of the microbiota differed at the OTU level between the control, CRC, CRC + Fn and CRC + Fn + Cys groups; the same was true for fungi (Fig. 6a, right). To further observe the abundance and diversity of microbiota in each group, Chao1 was used to measure the alpha diversity. For bacteria (Fig. 6b), the Chao1 showed that the abundance and diversity of microbiota were larger in the control group than in the other three groups. For fungi (Fig. 6c), when alpha diversity was measured using the Chao1 index, the CRC + Fn group and CRC + Fn + Cys group had lower alpha diversity than the control group. Principal coordinate analysis (PCoA) of the microbiota of the four groups showed that for bacteria (Fig. 6d, left), there were significant differences in the distribution of microbiota between the control group, CRC group, and CRC + Fn and CRC + Fn + Cys groups. However, the differences in the distribution of bacteria between the CRC + Fn and CRC + Fn + Cys groups were not significant. The PCoA (Fig. 6d, right) of the fungi showed that only the distribution of microbiota in the control group was different from the other three groups. The relative abundance of the top 10 bacteria at the genus level in the mouse intestine (Fig. 6e) was compared and clustered between groups, and it was found that the bacteria were specific between each group, e.g., *Escherichia_Shigella* in the CRC group was more abundant than the other groups, and *Lachnospiraceae_NK4A136_group* in the Fn group was more abundant than the other groups, but the fungi were not specific between each group. Fungi can be broadly classified into three groups, namely saprotroph, symbiotroph, and pathotroph, which obtain nutrients by degrading dead cells (saprotroph), exchanging nutrients with the host (symbiotrophs), and damaging host cells (pathotroph), respectively. Further analysis of the composition of fungi (Fig. 6f) showed that the abundance of fungi of the pathotroph group increased in the CRC and CRC + Fn groups when compared with that of the control group, while the abundance of fungi of the symbiotroph group decreased. However, there was no significant difference in the composition of fungi of pathotroph in the CRC + Fn + Cys group when compared with that of the control group. As seen from the linear discriminant analysis effect size (LFfSe) clustering diagram, there were some differences in the composition of intestinal bacteria (Fig. 7a) between the four groups. On the other hand, there were no significant differences in intestinal fungi (Fig. 7b) between the four groups from the clustering diagram. Taken together, these results indicate that Fn and H_2_S significantly reduce the alpha diversity and alter the beta diversity of the intestinal microbiota of mice, resulting in dysbiosis in the intestinal microbiota of mice.

In order to investigate which bacteria play a role in the promotion of CRC development by Fn and H_2_S, the bacteria in the four groups at the species level were analyzed (Fig. 6g). At the species level, in the control group, *unclassified_Muribaculaceae*, *unclassified_Prevotellaceae_NK3B31_group*, *unclassified_Prevotellaceae_UCG_001,* and *unclassified_Clostridia_UCG_014* were significantly more abundant than the CRC, CRC + Fn and CRC + Fn + Cys groups. In addition, the abundance of *unclassified_[Eubacterium]_coprostanoligenes_group* was significantly lower in the CRC + Fn and CRC + Fn + Cys groups. In the CRC group, it was found that *Escherichia_Shigella*, *Parasutterella* and *Clostridium*_*sensu*_*stricto*_*1* were significantly more abundant than in the other three groups. In the CRC + Fn group, it was found that *Lachnospiraceae*, *Lachnospiraceae*_*NK4A136*_*group,* and *Alistipes* were more abundant than in the other three groups. Finally, In the CRC + Fn + Cys group, *Odoribacter* and *Butyricicoccus* were more abundant than the other three groups.

Similarly, the fungi between the four groups were analyzed at the species level (Fig. 6h). It can be seen that the level of *unclassified_ Archaeorhizomyces* and *unclassified*_*Ascomycota* was significantly higher in the control group than that found in the CRC + Fn group, and *unclassified*_*Ascomycota* was also more abundant at the genus level than in the CRC + Fn group. In addition, the level of *Pichia*_*fermentans* in the control group was significantly higher than the other three groups, and there was a gradual decrease in the level of this fungus from the CRC group to the CRC + Fn + Cys group. Among them, *unclassified*_ *Archaeorhizomyces* is a saprophytic nutritional fungus and *Pichia*_*fermentans* is commonly found in fermented dairy products, which is a known beneficial fungus. The *unclassified*_*Candida* in the CRC + Fn group was significantly more abundant than that of the CRC group and the CRC + Fn + Cys group.

**Fig 8:**
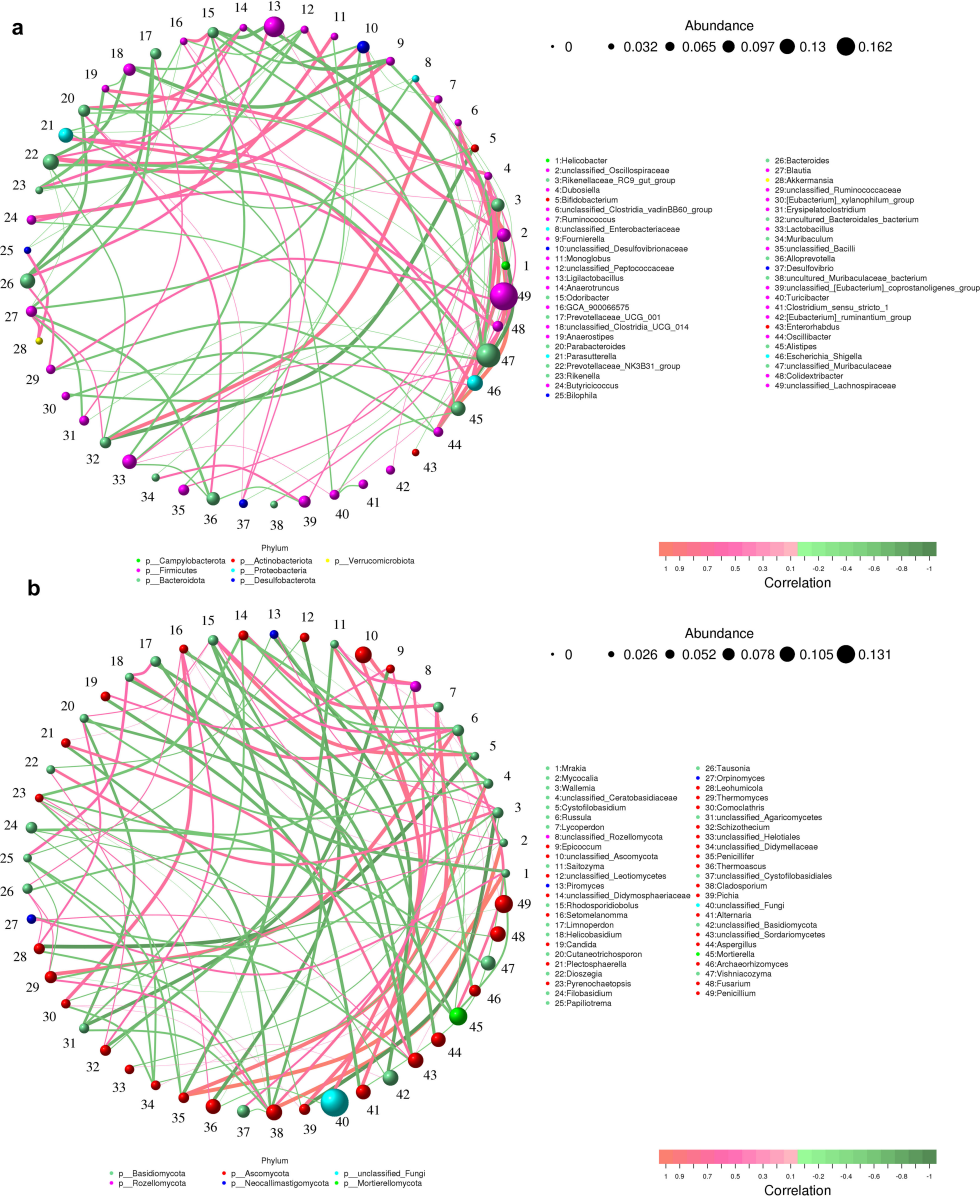
Correlation network plots for each species at the genus level for bacteria (a) and fungi (b).

As can be seen from the species correlation network plot (Fig. 8a), the abundance of *Lachnospiraceae* with high expression in the CRC + Fn group was negatively correlated with *Muribaculaceae*, *Prevotellaceae*_*NK3B31*_*group*, and [*Eubacterium*]_*coprostanoligenes*_*group*, which had higher expression in the control group, and was positively correlated with the abundance of *Lachnospiraceae*_*NK4A136*_*group* with high expression in the CRC + Fn group and *Rikenellaceae*_*RC9*_*gut*_*group* with high expression in CRC + Fn + Cys group. In addition, the abundance of *Lachnospiraceae*_*NK4A136*_*group* with elevated expression in the CRC + Fn group was positively correlated with *Odoribacter* and *Butyricicoccus* with elevated expression in the CRC + Fn + Cys group, suggesting a reciprocal promotion of harmful bacteria between the CRC + Fn and CRC + Fn + Cys groups. The *Muribaculaceae* with elevated expression in the control group was positively correlated with the higher expression levels of *Prevotellaceae*_*NK3B31*_*group*, *Clostridia*_*UCG*_*014*, [*Eubacterium*]_*coprostanoligenes*_*group,* and *Prevotellaceae*_*UCG*_*001*, which are all beneficial bacteria. Interestingly, *Muribaculaceae,* which is a probiotic, was positively correlated with *Alistipes*, an oncogenic bacterium with elevated expression in the CRC + Fn group, which may reveal the duality of *Muribaculaceae. Odoribacter* with elevated expression in the CRC + Fn + Cys group was positively correlated with *Desulfovibrio*, which is capable of producing H_2_S, and *Lachnospiraceae* in the CRC + Fn group. In addition, it was negatively correlated with the probiotic *Bifidobacterium*.

At the level of fungal genera (Fig. 8b), the abundance of *unclassified*_*Ascomycota* was higher in the control group, which was positively correlated with the probiotic *Archaeorhizomyces* and also significantly positively correlated with *Russula. Archaeorhizomyces* was negatively correlated with *Lycoperdon*, but positively correlated with *Sebacina. Pichia*_*fermentans* was positively correlated with *Wallemia*, *unclassified*_*Ceratobasidiaceae*, *unclassified*_*Leotiomycetes*, *Plectosphaerella*, *Gibellulopsis*, *Hanseniaspora*, and *Arthrobotrys*. These results reveal that a certain pathogenic or beneficial bacterium or fungus has an immediate effect on the abundance of other surrounding bacteria or fungi, and that harmful bacteria or fungi are often negatively correlated with beneficial bacteria or fungi and vice versa with harmful bacteria or fungi.

As seen from KEGG function analysis (Fig. 6i), more functional pathways were changed in the CRC + Fn group and CRC + Fn + Cys group than in the CRC group when compared with the Control group. The membrane transport pathway was enhanced in the CRC + Fn group when compared to that of the control group, while the metabolism of vitamins and cofactors, cellular transcription process, polysaccharide synthesis, and metabolism pathways were all diminished. The CRC + Fn + Cys group showed attenuated transcription and translation processes when compared to the control group, in addition to attenuated metabolism of nucleotides, vitamins, and cofactors.

## DISCUSSION

H_2_S has long been considered to be harmful to the body, and in recent years, its twofold nature has been continuously reported. On the one hand, H_2_S protects bacteria from host immune system attack and is involved in the DNA damage response (18, 19); however, on the other hand, it also has antioxidant effect, mitochondrial protection, and ion channel regulation functions (20, 21). The approximate concentration of H_2_S in the human colon has been reported to be 250 µmol/L (22), and the concentration of H_2_S in the colon has also been reported to be 0.3–3.4 mmol/L or 0.1–2.0 mmol/L (23). Levine et al. (24)reported that the amount of hydrogen sulfide in the feces of patients with colitis was about four times higher than that found in fecal samples taken from people not suffering from bowel disorders. In mammals, there are three enzymes known to produce H_2_S, namely cystathionine-β-synthase (CBS), cystathionine-γ-lyase (CSE) and 3-mercaptopyruvate sulfurtransferase (3-MST). A growing body of research suggests an inextricable link between H_2_S and cancer. In many cancers, including CRC, all three enzymes become highly expressed. One such example of this is in CRC, the upregulation of CBS is particularly pronounced. Simultaneously, tumor cells up-regulate their ability to produce H_2_S in order to supply the energy needed for growth (20). Fn is a bacterium that normally resides in the oral cavity and metabolizes Cys to produce H_2_S; this bacterium is often detected in the fecal samples obtained from CRC patients. Although many studies have associated H_2_S with CRC or Fn with CRC, few studies have reported the role of H_2_S metabolized by Fn in the development of CRC. In the above study, the concentration of H_2_S that promoted proliferation and migration of CRC cell lines was investigated, and it was combined with the concentration of H_2_S in the colon of CRC patients reported in the literature in order to deduce the concentration of Cys to be added. Previous studies have reported that Fn might promote tumor metastasis and generate resistance to chemotherapy by activating the autophagic pathway (14, 25, 26). Thus, HCT116 cells were co-cultured with Fn or Fn +Cys for a period of time and the changes in genes and pathways were investigated, especially autophagy-related genes and oncogenic pathways, by sequencing the cellular transcriptome. Meanwhile, by giving Fn or Fn +Cys to AOM/DSS model mice, the changes of autophagy markers and inflammatory factors in serum and tissues of mice were observed. It was clearly demonstrated by the ELISA and RT-qPCR analyses that Fn did indeed promote the increase of inflammatory factors and autophagy in cancer cells and mice, while H_2_S further promoted autophagy. Furthermore, 16S rRNA and ITS obtained from the feces of mice were sequenced and it was found that Fn and H_2_S dysregulated intestinal microbiota by promoting the growth of harmful bacteria and inhibiting the growth of beneficial bacteria.

Since pathological concentration of H_2_S promotes the development of CRC, it is possible that the use of some inhibitors of H_2_S may have a therapeutic effect on CRC. For example, some inhibitors of H_2_S-producing enzymes are commonly used, such as CSE-specific inhibitor: propargylglycine; CBS-specific inhibitor: amino-oxyacetic acid; and the 3MST-specific inhibitor: aspartic acid. However, these inhibitors are known to cause harm to normal cells. Li et al. (27) achieved the elimination of H_2_S by using anti-tumor nanomaterials FeOOH NSs, which had the advantages of low toxicity and high specificity.

In transcriptome sequencing of HCT116 cells, it was found that among the genes co-up-regulated in the HCT116 + Fn group and the HCT116 + Fn + Cys group, TNF is involved in AOM/DSS-induced colorectal carcinogenesis and is closely related to bacterial abundance. No upregulation of *ARMH2* has been reported in colorectal patients. *MAP1LC3A* is a gene that encodes a key autophagic protein in the process of autophagy. Gil et al. found that the level of *MAP1LC3A* was significantly lower in CRC tissues than in adjacent non-cancerous tissues by RT-qPCR analysis (28), which contradicts the cellular transcriptional results reported in this study.

Among the genes that increased after HCT116 cells were administered with only Fn, Liu et al. (29) demonstrated that *ACTC1* was overexpressed in cancer tissues when compared to the paracancerous tissues of epithelial ovarian cancer and, together with *GRK5*, constitutes a biomarker of prognosis. *PCP4* is often expressed together with Purkinje cell peptide (PEP) as a peptide in breast cancer, and they inhibit apoptosis of breast cancer cells while enhancing migration and adhesion of cancer cells (30). In human or carious bladder tumors, the expression of *PCP4* shows a decreasing trend, suggesting that the role of *PCP4* is different in different cancers. But at present, there has not been a study completed on the association between *PCP4* and colorectal cancer. *IL13RA2* is expressed in nearly 80% of lung cancers (31), and its expression is also elevated in colorectal cancer sites when compared to that of normal sites. In addition, among the genes that decreased, metallothionein (MT) is a cysteine-rich protein that is divided into many isoforms, MT1B is one of them. *MT1B* has been reported to be down-regulated in colorectal cancer and is associated with poor clinical outcome; however, this gene is up-regulated in other cancers, such as non-small cell lung cancer (32).

After administering Fn and Cys to HCT116, some genes increased. For example, in several metastatic cancers, such as colorectal, breast, and prostate cancers, *SELE* expression increased when compared to that of healthy populations (33). *CCL20* induced proliferation and migration of colorectal cancer epithelial cells (34). *CXCL11* expression was significantly elevated in both colon adenocarcinoma and rectal adenocarcinoma; thus, *CXCL11* could be used as a marker of colon adenocarcinoma (35). There were also some genes that were decreased. Nandy et al. (36) again verified that *SFTPD* with elevated serum expression level was associated with chronic obstructive pulmonary disease and *MS4A12* was a novel colon cancer-associated factor, the expression of this gene was dependent on CDX2.

Fn and its metabolite H_2_S also exerted significant effects on the microbiota in mice. Chung et al. (37) demonstrated that *Muribaculaceae* were the main families of bacteria at the family level inhabiting the intestines of healthy mice, and that bacteria such as *Lachnospiraceae* became the main bacteria in the intestine of mice given AOM/DSS to induce colon cancer. These changes in bacterial abundance are consistent with the experimental results reported in this study. An assay on the dynamics of the intestinal microbiota in mice with ulcerative colitis showed that *Clostridia_UCG_014* increased first on day 14 after modeling and decreased on day 21, and *Muribaculaceae* decreased to 6.22% of normal levels in the LPS-induced intestinal injury model (38), both suggesting that *Muribaculaceae* is negatively associated with intestinal inflammation in mice. Hu et al. (39) showed a significant increase in the abundance of beneficial bacteria such as *Prevotellaceae_NK3B31_group* and a significant decrease in the abundance of CRC-associated bacteria at the genus level after administration of diallyl disulfide, a component of garlic, in DSS-molded mice. *Prevotellaceae_UCG_001* can metabolize inulin to produce short-chain fatty acids and is a known beneficial bacterium. In genetically deficient obese mice, the abundance of cecum *Prevotellaceae_UCG_001* was negatively correlated with the levels of liver index, serum total cholesterol, and high-density lipoprotein cholesterol (40). The abundance of *Clostridia_UCG_014* decreased in the LPS-induced intestinal injury mouse model and recovered after probiotic treatment, while the abundance of *Escherichia-Shigella* and *Lachnospiraceae_NK4A136*, two harmful bacteria, decreased after probiotic administration (38). *Eubacterium_coprostanoligenes* is also a probiotic that modulates dyslipidemia in high-fat diets and may exert some regulatory effects on host lipid metabolism (41). In addition, the abundance of *Lactobacillus_iners* was significantly higher in the control group than in that of the CRC and CRC + Fn groups, but this difference was not observed in the CRC + Fn + Cys group. Taken together, these data suggest that Fn and its metabolite H_2_S promote CRC by decreasing the abundance of some probiotic bacteria.

On the other hand, the number of harmful bacteria in the model group increased. *Parasutterella* and *Clostridium*_*sensu*_*stricto*_*1* became dynamic over time in the mouse model of colitis, i.e., they first increased and then decreased, and both bacteria were positively correlated with the inflammatory factors TNF-α, IL-4, interferon gamma (IFN-γ), and 5-hydroxytryptamine (5-HT) at serum levels, but negatively correlated with the anti-inflammatory factors IL-22 and IL-10 (42), which is consistent with the experimental results obtained in this study. Cao et al. (43) found that bacteria such as *Parasutterella* and *Odoribacter* had a significant decrease in the 1.5% DSS-induced colitis mouse model, while the abundance of both bacteria recovered after treatment. This is contradictory to the experimental results in this study, because we had a significant increase in *Parasutterella* in the CRC group when compared to that of the control group after the administration of AOM/DSS-induced colitis; *Odoribacter* in the CRC + Fn + Cys group also showed a significant increase when compared to the other three groups.

Chung et al. (37) found that *Lachnospiraceae* became one of the dominant bacteria at the family level after AOM/DSS induction of colorectal cancer in mice. They postulated that some members of *Lachnospiraceae* were associated with ecological balance in the intestine. In addition, another study (42) showed that *Lachnospiraceae*_*NK4A136*_*group* increased and then decreased in a mouse model of colorectal cancer and that this bacterium was positively correlated with the levels of inflammatory cytokines IL-6 and IL-22, which corroborated the experimental findings of this study. Hua et al. (44) identified *Alistipes* and *Odoribacter* as the core bacteria present in colorectal adenoma-cancer by 16S rRNA gene sequencing of mouse feces, indicating the important role played by these two bacteria in colorectal cancer, which is consistent with this study. Disturbance in lipid metabolism is an important factor in CRC, and intestinal microbiota are involved in lipid metabolism. *Butyricicoccus*, a butyric acid-producing bacterium, was shown, in a clinical study (45), to be present in CRC patients without hyperlipidemia and hypercholesterolemia. In addition, its abundance in the fecal samples of patients with advanced CRC was lower than in the fecal samples obtained from patients with early disease, i.e., the abundance of *Butyricicoccus* decreased gradually as CRC progressed, this differs from the experimental results of this study. In terms of fungi, the beneficial or harmful effects of Candida have to be analyzed on a case-by-case basis, for example, *Candida*_*versatilis* and *Candida_sp_JCM_15000* are mainly found in healthy populations (46), whereas *Candida albicans* is an opportunistic pathogen that can promote the development of CRC (47). In general, our results illustrate that Fn and its metabolite H_2_S promote CRC by increasing the abundance of harmful bacteria and fungi as well as decreasing the abundance of beneficial bacteria and fungi.

Autophagy, like H_2_S, has a double-sided effect. Chloroquine is an inhibitor of autophagy, while rapamycin is an agonist of autophagy. Sena et al. (48) suggested that inhibition of autophagy could be a potential target for the treatment of colorectal cancer. Harbaum et al. (49) demonstrated that chloroquine, an autophagy inhibitor, can inhibit CRC metastasis by reducing Fn-induced overexpression of KRT7, both of which means autophagy may promote the progression of CRC (10). In detail, Fn can induce autophagy process in CRC cells, thus improving the resistance of CRC cells to chemotherapy (10). In this study, the main autophagy-related genes included *DRAM1*, *NBR1*, *ATG7*, *MAP1LC3A*, and *ATG16L1*. ATG7 and ATG5 are two key proteins that control autophagosome formation and are closely related to the occurrence of autophagy. Deficiency of ATG7 in tumor cells but not in normal cells led to p53-mediated cell cycle arrest, suggesting a pro-oncogenic role for this gene (50). In CRC tumors with high microsatellite instability, DRAM1 shows high expression (51) and it plays a positive regulatory role in the autophagy process (52). In addition, *DRAM1* expression decreased in human colon cancer cells treated with anti-PD-1 nivolumab, suggesting that *DRAM1* is a pro-cancer-associated autophagy gene (53), which is consistent with the results of this study. NBR1, one of the autophagy receptors, is able to recruit autophagy proteins that promote autophagosome formation and facilitate the process of autophagy (54). In human urothelial cancer cells, *NBR1* shows high expression, and *NBR1* is widely involved in the migration and development of various tumors, such as breast cancer and pancreatic cancer (55). MAP1LC3A is also an autophagy-associated protein that has been reported to be involved in the process of autophagy and is involved in the development of breast cancer (56). The allele Thr300Ala in *ATG16L1* is strongly correlated with increased overall survival in human CRC (57), which may explain why *ATG16L1* decreased rather than increased in the CRC + Fn + Cys group.

Although this study revealed that Fn and its metabolite H_2_S altered gut microbiota composition and autophagy process in CRC as well as promoted CRC progression, the specific pathways through which H_2_S promotes autophagy and the specific mechanisms by which autophagy-related genes regulate the development of colorectal cancer were not investigated in this study, which required further research.

## MATERIALS AND METHODS

### Bacterial strain and culture

*F. nucleatum* subsp. *nucleatum* ATCC 25586 was purchased from Guangdong Microbial Culture Collection Center; it was cultured on solid agar plates made with Brain Heart Infusion Broth (QDRS BIOTEC, cat. no. 10805) under anaerobic conditions at 37°C. Liquid cultures were grown in a Tryptic Soy Broth medium (Qingdao Hope Bio-Technology Co Ltd) under anaerobic conditions at 37°C.

### Culture of cancer cells

Two human colon cancer cell lines, RKO ATCC CRL-2577 and HCT116 ATCC CCL-247, and one human colorectal cancer epithelial cell, DLD1 ATCC CCL-221, were cultured in RPMI-1640 medium (ThermoFisher) supplemented with 10% fetal bovine serum (Shanghai yuye Bio-Technology Co Ltd) and incubated in 5% CO_2_ and 95% air at 37°C.

### Co-culture of *F. nucleatum* with cancer cells

Single colony of bacteria was picked from tryptic soy agar plate and inoculated into Trypticase soy broth (TSB) medium cultured overnight, and the absorbance was measured at 600 nm so that the absorbance was about 0.041 (i.e., the number of bacteria was about 10^7^). The bacteria were washed with phosphate buffered saline (PBS) and re-suspended in a sterile and oxygen-free PBS solution before the experiment. For infections, cells were passaged the day before the experiment, and the cells were inoculated in six-well plates. When the number of cells was 2.5 × 10^5^, three groups were set up with three parallel wells in each group: blank control, cells + bacteria, cells + bacteria + L-cysteine group with 200 µL PBS, 100 µL PBS solution of bacteria and 100 µL PBS, 100 µL PBS solution of bacteria and 100 µL L-cysteine hydrochloride solution, respectively. Fn was used at multiplicity of infection (MOI) of 100:1, and HCT116 was incubated with Fn for 4 h at 37°C with 5% CO_2_ (58).

### RNA-seq transcriptome

Total RNA was extracted from the cells using TRIzol Reagent (Vazyme). In detail, after 4 h of cell culture, the cell culture fluid was aspirated. TRIzol was added at a ratio of 2 mL TRIzol per 10 cm^2^ well area and slowly rotated for approximately 10 min to ensure total exposure and digestion of TRIzol and cells. Finally, the cytosol containing TRIzol was transferred to RNase-free tubes, and the cells were repeatedly blown with a gun to fully dissolve in TRIzol and stored at −80°C. Then RNA-seq transcriptome libraries and cDNA were prepared. Finally, the DEGs (differential expression genes) between two samples were selected and GO functional enrichment and KEGG pathway analysis were conducted to investigate the functions of the DEGs.

### CCK-8 assay

RKO, HCT116, and DLD1 cells were set up in four groups, respectively, with five wells in parallel in each group. The absorbance per well was first measured at 450 nm with CCK-8 reagent using spectrophotometer (Bio-Walker, China) when NaHS (MREDA TECHNOLOGY INC, cat. no. M02663-100g) was not added (i.e., 0 h) and recorded as OD_0h_. Then, NaHS solution was added to the four groups so that the final concentration of NaHS was 0, 0.2, 1, and 2 mM, respectively, and the culture medium was used as blank; then, the cells were incubated in 96-well plates for 24 h. CCK-8 reagent was added, and the absorbance per well was measured at 450 nm 2 h later and recorded as OD_24h_. The relative survival of cells was calculated as follows: (%) = (OD_24h_/OD_0h_) × 100%

### Cell scratch assay

RKO, HCT116, and DLD1 cells were seeded in 12-well plates and left to grow until confluent. After wounding with 200 µL sterile pipette tips, the cells of each well were incubated with different concentrations of NaHS and cell images were taken at 0, 24, and 48 h.

### Detection of hydrogen sulfide concentration

Detection of hydrogen sulfide in culture medium was performed by the bismuth chloride method (59). Simply, 100 µL of freshly prepared bismuth solution and an equal volume of bacterial culture solution (supernatant or PBS solution of bacteria) or cell culture solution were added sequentially in a 96-well plate, and a clear color change was visible in the presence of hydrogen sulfide. The absorbance was measured at 405 nm.

The total hydrogen sulfide content in mouse feces was measured by a slightly adjusted methylene blue method (60, 61). Briefly, mouse feces were homogenized with anaerobic NaCl, followed by the addition of zinc acetate (Sinopharm Chemical Reagent Co., Ltd.) solution (which can be stored at −20°C). Then N,N-dimethylphenyl hydrochloric acid solution (MACKLIN) and ferric chloride hydrochloric acid solution (Sinopharm Chemical Reagent Co., Ltd.) were added sequentially, placed at 35°C for 35 min, and centrifuged to remove turbidity, and 50 μl of the supernatant was added to 1 ml of 0.86 M Tris-Hcl buffer (pH=0.6). Finally, 100 µL was taken to measure the absorbance at 670 nm.

### Treatments of animals

C57BL/6N male mice at 6 weeks of age were divided into four groups: control (i.e., wild-type mice), CRC, CRC + Fn, and CRC + Fn + Cys, with eight mice in each group. For CRC, CRC + Fn and CRC + Fn + Cys groups, 2 mg/mL streptomycin and 0.8 mg/mL penicillin were first given in drinking water for 1 week. After which, the CRC model was created: the three groups above were given azoxymethane (12 mg/kg, aladdin) intraperitoneally on day 1 and 2% (wt/vol) dextran sulfate sodium (Meilunbio) in drinking water for 7 consecutive days starting on day 6, followed by 2 weeks of regular drinking water. This was followed by two cycles of “7 consecutive days of DSS and 2 weeks of regular drinking water.” In addition, during the modeling period, the CRC + Fn group was gavaged with Fn (10^9^ CFU) every 2 days; the CRC group was gavaged with PBS every 2 days; and the CRC + Fn + Cys group was gavaged with L-cysteine (100 mg/kg, scientan) once a day per mouse during the “two weeks of regular drinking water” period, in addition to Fn every 2 days until the end of the experiment. Mice weight was recorded approximately every 3 days (twice a week).

Mice were sacrificed on the 70th day of modeling; colons were collected to analyze the number and size of tumors, then they were fixed in 4% paraformaldehyde for 3 to 4 days, paraffin-embedded, and H&E staining was used to observe histology. The rest of the tissues were frozen at −80°C for ELISA and RT-qPCR; blood of mice was collected and left for 30 min and then centrifuged at 3,000 rpm for 10 min. The upper layer of serum was collected, and the autophagy and inflammatory factors were detected by ELISA. Mice feces were collected for 16S rRNA and ITS sequencing to detect the composition and function of bacteria and fungi among different groups, respectively.

### Detection of inflammatory cytokines by ELISA

The level of NLRP3, IL-1β, p62, and LC3II in the serum of the mice was evaluated using Mouse NLRP3, IL-1β, p62, LC3II ELISA kit (SinoBestBio, China), respectively. The level of VEGF, COX-2, IL-6, and cadherin-17 in the colon of the mice and cells was evaluated using VEGF, COX-2, IL-6, CDH-17 ELISA kit (SinoBestBio, China), respectively.

### RT-qPCR for the detection of autophagy-related genes

Total RNA was extracted using TRIzol reagent (Vazyme) according to the manufacturer’s instructions. A HiScript II One Step qRT-PCR SYBR Green Kit (Vazyme, China) was used to perform RT-qPCR under the following conditions: reverse transcription at 50℃ for 3 min, initial denaturation at 95℃ for 30 s, 40 cycles of cyclic reaction at 95℃ for 10 s and 60℃ for 30 s, melting curve at 95℃ for 15 s, 60℃ for 60 s, and 95℃ for 15 s. The primer sequences were as follows (Table 5):

**TABLE 5:** Primer sequences of RT-qPCR

Gene	Primer sequences（5´−3´）
DRAM1	Forward： CTCTGCATTTCTTGGCGCAG
	Reverse：AACGGGAGTGCTGAAGTAGC
NBR1	Forward： AAAGCACCTCCTGGCTTTGT
	Reverse： AAGCAGGGGTCTCAGCTTTC
ATG7	Forward： CACAGTGGTGAGGCCAACTC
	Reverse： ACTGTTCTTACCAGCCTCACTG
MAP1LC3A	Forward：GGTCCTGGCTCCTAAACTAAG
	Reverse：AAAAGAGCAACCCGAACAT
ATG16L1	Forward： TTCTGATGCTGCCAGGAGAC
	Reverse: ACTATTTCTCTAGTGCCTGAAGAC
GAPDH	Forward: CGCTAACATCAAATGGGGTG
	Reverse: TTGCTGACAATCTTGAGGGAG

### Statistical analysis

All experiments were repeated at least three times in parallel. Data were presented as mean ± SEM and were analyzed using GraphPad Prism 8 software. The differences in the data were evaluated by the Student’s *t*-test or analysis of variance.

## Data Availability

All the 16S rRNA and ITS sequencing data were submitted to the National Center for Biotechnology Information (accession number PRJNA905704). All the RNA sequencing data were submitted to the National Center for Biotechnology Information (accession number PRJNA925233). The other data of this study are available on request from W.C.
